# 2521 Use of forced air warming devices to induce fever-range hyperthermia in critically ill septic patients

**DOI:** 10.1017/cts.2018.191

**Published:** 2018-11-21

**Authors:** Anne M. Drewry, Enyo A. Ablordeppey, Marin H. Kollef, Richard S. Hotchkiss

**Affiliations:** 1 Department of Emergency Medicine, Washington University in St. Louis; 2 Department of Medicine, Washington University in St. Louis; 3 Department of Anesthesiology, Washington University in St. Louis

## Abstract

OBJECTIVES/SPECIFIC AIMS: Afebrile septic patients are twice as likely to die and develop nosocomial infections as compared with those with fever; the reason for these differences is unknown. One hypothesis is that elevated temperatures directly boost immunity and inhibit microorganism growth. However, there is little data examining the clinical effects of warming septic patients. The goal of this study was to determine whether warming afebrile septic patients to fever-range hyperthermia with noninvasive forced air warmers is feasible and safe. METHODS/STUDY POPULATION: This is an ongoing randomized trial on afebrile mechanically ventilated patients with severe sepsis. The intervention consisted of 48 hours of external warming with a forced air warming device to a goal core temperature of 1.5°C higher than the lowest recorded temperature within the 24 hours preceding enrollment. Efficacy of the intervention and adverse event data (i.e., increases in heart rate and vasopressor doses) were collected. Clinical outcomes included 28-day mortality and acquisition of secondary infections. RESULTS/ANTICIPATED RESULTS: In total, 18 patients were randomized to the control and warming groups, respectively. Baseline characteristics (including demographics, comorbidities, and illness severity scores) were similar among the 2 groups, except the control group had more males (61% vs. 28%, *p*=0.04). Median (IQR) body temperature averaged over the 48-hour intervention period was higher in the warming group [38.2 (37.6, 38.6) vs. 37.1 (36.4, 37.4) °C, *p*<0.001). Patients in the warming group achieved core temperatures above their goal for a median of 37 (IQR 11, 45) hours during the 48-hour intervention period. There were no differences in heart rate or vasopressor dose changes or acquisition of secondary infections between the groups. Eight (44.4%) control patients and 3 (16.7%) warmed patients died by day 28 (*p*=0.07). DISCUSSION/SIGNIFICANCE OF IMPACT: Externally warming severe septic patients with forced air warming devices effectively raises core body temperature and is safe. Additional research will focus on cellular and immunological changes seen in warmed Versus control patients.

